# Supplementary material for: Is a Combination of Metals More Toxic to Mosses Than a Single Metal?
Source: Plants (Basel). 2023 Nov 24;12(23):3960. doi: 10.3390/plants12233960 (PMC10708001; doi:10.3390/plants12233960)
Supplement: Supplementary file 1 [file plants-12-03960-s001.zip › plants-2717782-supplementary.pdf]

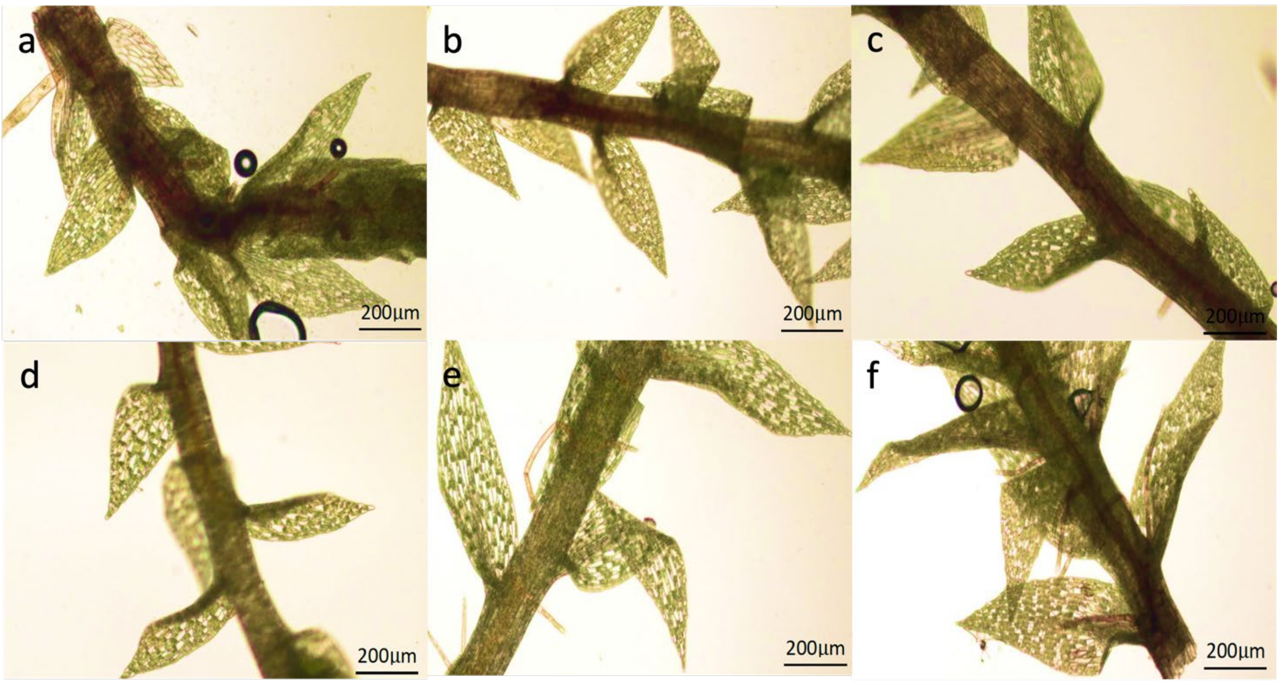

Figure S1: Plasmolysis test for *P. drummondii* for combination of two metals in solution at 100 µM. a: $CuCl_2$+$FeCl_2$; b: $CuCl_2$+$MnCl_2$; c: $CuCl_2$+Sb-Acetate; d: $MnCl_2$+$FeCl_2$; e: $MnCl_2$+Sb-Acetate; f: $FeCl_2$+Sb-Acetate

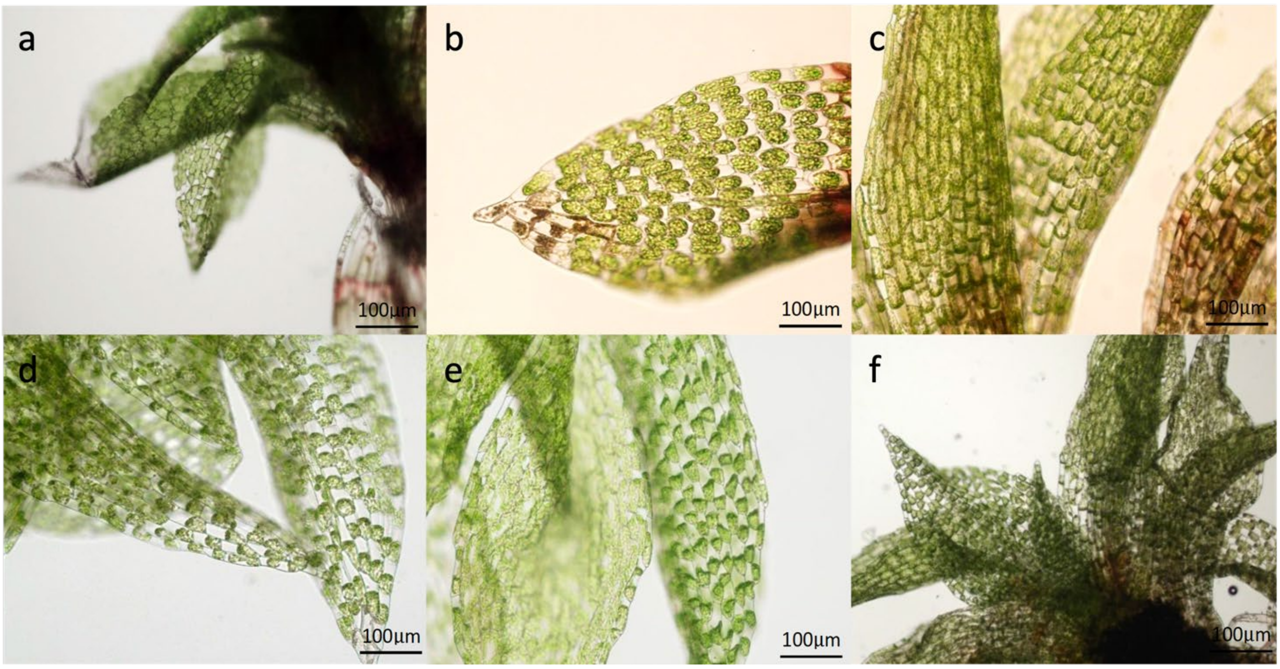

Figure S2: Plasmolysis test for *P. patens* for combination of two metals in solution at 100 µM. a: $CuCl_2$+$FeCl_2$; b: $CuCl_2$+$MnCl_2$; c: $CuCl_2$+Sb-Acetate; d: $MnCl_2$+$FeCl_2$; e: $MnCl_2$+Sb-Acetate; f: $FeCl_2$+Sb-Acetate

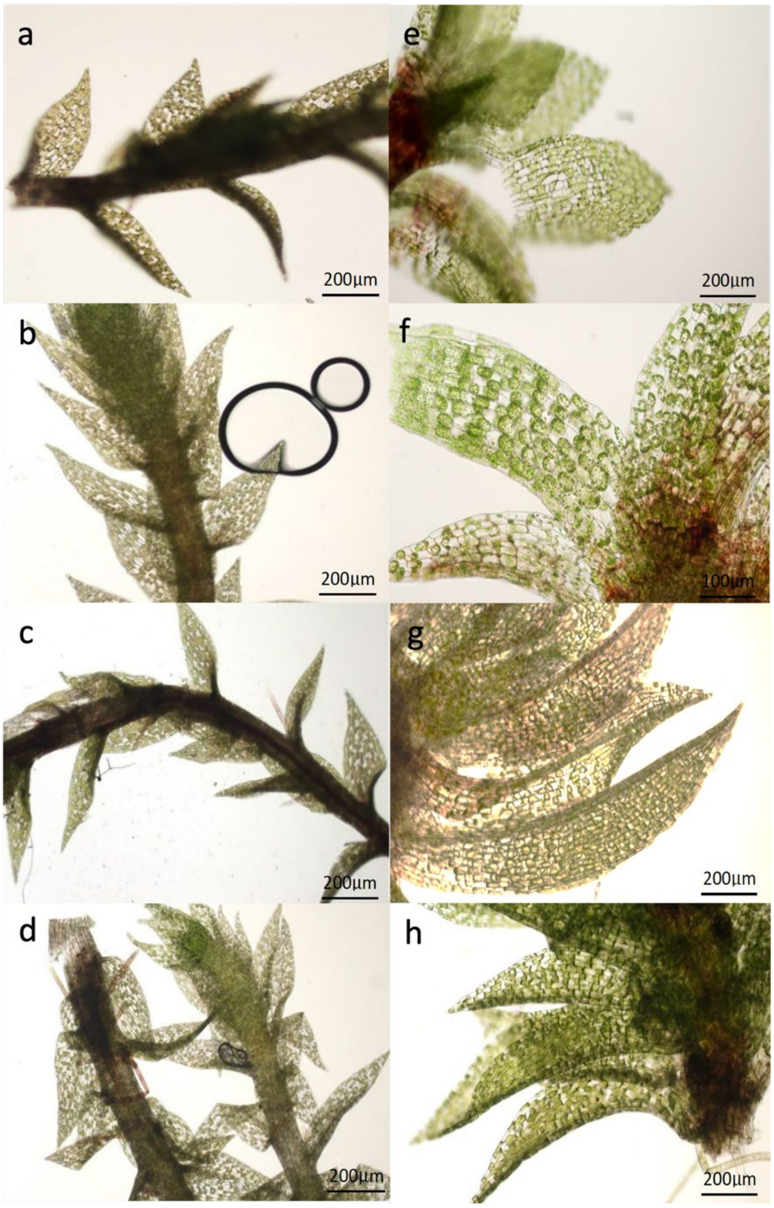

Figure S3: Plasmolysis test for *P. drummondii* (a, b, c, d) and *P. patens* (e, f, g, h) for combination of three metals in solution at 100 µM. a: $CuCl_2$+$MnCl_2$+$FeCl_2$; b:$CuCl_2$+$MnCl_2$+Sb-Acetate; c: $CuCl_2$+$FeCl_2$+Sb-Acetate; d: $MnCl_2$+$FeCl_2$+Sb-Acetate; e: $CuCl_2$+$MnCl_2$+$FeCl_2$; f: $CuCl_2$+$MnCl_2$+Sb-Acetate; g: $CuCl_2$+$FeCl_2$+Sb-Acetate; h; $MnCl_2$+$FeCl_2$+Sb-Acetate.
